# Intronic and plasmid-derived regions contribute to the large mitochondrial genome sizes of Agaricomycetes

**DOI:** 10.1007/s00294-014-0436-z

**Published:** 2014-07-11

**Authors:** Kajsa Himmelstrand, Åke Olson, Mikael Brandström Durling, Magnus Karlsson, Jan Stenlid

**Affiliations:** Department of Forest Mycology and Plant Pathology, Uppsala BioCenter, Swedish University of Agricultural Sciences, Box 7026, 750 07 Uppsala, Sweden

**Keywords:** Mitochondrial genome, Mitochondrial evolution, Pathogen, Homing endonuclease genes, Plasmid DNA

## Abstract

Sizes of mitochondrial genomes vary extensively between fungal species although they typically contain a conserved set of core genes. We have characterised the mitochondrial genome of the conifer root rot pathogen *Heterobasidion irregulare* and compared the size, gene content and structure of 20 Basidiomycete mitochondrial genomes. The mitochondrial genome of *H. irregulare* was 114, 193 bp and contained a core set of 15 protein coding genes, two rRNA genes and 26 tRNA genes. In addition, we found six non-conserved open reading frames (ORFs) and four putative plasmid genes clustered in three separate regions together with 24 introns and 14 intronic homing endonuclease genes, unequally spread across seven of the core genes. The size differences among the 20 Basidiomycetes can largely be explained by length variation of intergenic regions and introns. The Agaricomycetes contained the nine largest mitochondrial genomes in the Basidiomycete group and Agaricomycete genomes are significantly (*p* < 0.001) larger than the other Basidiomycetes. A feature of the Agaricomycete mitochondrial genomes in this study was the simultaneous occurrence of putative plasmid genes and non-conserved ORFs, with *Cantharellus cibarius* as only exception, where no non-conserved ORF was identified. This indicates a mitochondrial plasmid origin of the non-conserved ORFs or increased mitochondrial genome dynamics of species harbouring mitochondrial plasmids. We hypothesise that two independent factors are the driving forces for large mitochondrial genomes: the homing endonuclease genes in introns and integration of plasmid DNA.

## Introduction

Mitochondria are believed to have a monophyletic origin from an endosymbiotic α-proteobacterium that was engulfed, more than one billion years ago, by a eukaryotic common ancestor (Gray et al. 2001; Bullerwell and Lang 2005; Koumandou et al. 2013). The mitochondrial (mt) genomes contain considerably fewer genes than free-living α-proteobacteria and many of the genes that are required for its function seem to have been transferred to the nucleus or replaced by already existing nuclear genes with similar function (Adams and Palmer 2003). Mitochondrial genomes occur either as circular or linear molecules (Burger et al. 2003).

Genes encoded by the mt genome can be divided into two groups: core genes and exchangeable genes. Core genes are involved in respiration, oxidative phosphorylation and translation (Burger et al. 2003), while exchangeable genes are characterised by variation in type and number among species. There are relatively few differences of mt core genes between and within kingdoms and phyla, although the exact compositions can vary (Adams and Palmer 2003). The exchangeable genes can be intronic genes, plasmid-derived genes and non-conserved open reading frames (ORFs). Mitochondrial plasmids are commonly found in plant and fungal mitochondria and are self-replicating genetic elements that have little or no homology to the mt DNA, hence thought to have a separate evolutionary history from their hosts (Cahan and Kennell 2005; Formighieri et al. 2008). In Basidiomycetes, mt plasmids have been found in for example *Flammulina velutipes* (Nakai et al. 2000) and *Pleurotus ostreatus* (Yui et al. 1988) and integrated in mt genomes in *Agaricus bisporus* (Ferandon et al. 2013), *P. ostreatus* (Wang et al. 2008) and *Moniliophthora perniciosa* (Formighieri et al. 2008). The core genes in fungal mt genomes often contain introns with conserved RNA secondary structures involved in autocatalytic splicing (Lang et al. 2007). Many of these introns contain ORFs encoding homing endonuclease genes (HEGs) that can insert the intron into another intronless gene through a mechanism where the target gene is cleaved at rare recognition sites (Lang et al. 2007). This mechanism is called intron homing and could be the reason for the great variability of intron number between species. Some of the HEGs are also functioning as maturases that promote the RNA folding of the introns (Belfort 2003).

Mitochondrial sizes vary extensively in animals and plants with fungal mt sizes being intermediate (12–236 kb). At present, there are annotated mt genomes from 165 fungal species (http://www.ncbi.nlm.nih.gov/genomes/GenomesGroup.cgi?opt=organelle&taxid=4751&sort=Genome). The fungal species investigated so far have a core set of 13–15 protein coding genes and two rRNA genes (*rns*, *rnl*) in common, although *atp9* are lost in euascomycetes (Adams and Palmer 2003). Two other genes, *rps3* and *rnpB*, are repeatedly lost in various fungal lineages (Adams and Palmer 2003; Bullerwell and Lang 2005). Seven genes that encode subunits of the NADH dehydrogenase complex are lost in the mt genomes of the genus *Schizosaccharomyces* and some *Saccharomyces* species (Bullerwell and Lang 2005).

In addition to energy production, the mitochondria play a fundamental role in metabolism, ion homeostasis, and apoptosis (Burger et al. 2003). The mitochondrion can also have a role in altering the virulence of fungal pathogens (Rogers et al. 1987; Monteiro-Vitorello et al. 1995; Ghabrial and Suzuki 2009; Ma et al. 2009; Monteiro-Vitorello et al. 2009). We have earlier shown that the mitochondrial origin influence virulence in *Heterobasidion* hybrids (Olson and Stenlid 2001).

*Heterobasidion irregulare* belong to the *H. annosum* sensu lato (s.l.) species complex in the phylum Basidiomycota, in the class of Agaricomycetes. Members of *H. annosum* s.l. are severe necrotrophic root pathogens on conifers, with different but partially overlapping host ranges (Dalman et al. 2010). *H. irregulare* is distributed throughout the temperate regions of North America and has several *Pinus* and *Juniperus* tree species as its main hosts (Otrosina and Garbelotto 2010). The nuclear and mitochondrial genomes of *H. irregulare* strain TC 32-1 were recently sequenced (Olson et al. 2012).

The first objective of this study was to characterise the mt genome of *H. irregulare* and to describe its unique features. The second objective was to perform a comparative Basidiomycete mt genome study, to investigate the processes involved in the large variations of mt genome sizes in Basidiomycetes.

## Materials and methods

### Genome annotation and analysis

The *H. irregulare* strain TC 32-1 genome v.2 (http://genome.jgi-psf.org/Hetan2/Hetan2.home.html) was used in the current work (Olson et al. 2012). The whole mt genome was obtained in one of the assembled contigs. Circularity was tested by performing several different assemblies that resulted in different overlapping start and end points of linear contigs. ORFs longer than 150 bp were identified using ORFfinder, codon usage Table 4 (http://www.ncbi.nlm.nih.gov/projects/gorf/). ORFs were used to search the non-redundant NCBI database with BLASTP to annotate and find conserved genes.

Putative plasmid genes were defined as genes with similarity (*E* value ≤0.002) to mitochondrial plasmid genes (integrated in mt genomes or not). Exon/intron boundaries of core genes were located by means of CLUSTALW alignment with homologous genes from other closely related fungal species. Predicted gene products from ORFs, 420 bp or longer, with limited similarity (*E* value >0.1) to characterised proteins were categorised as non-conserved ORFs (ncORFs). The ORFs were required to start with a methionine codon. ORFs with lengths 150–420 bp, not overlapping with annotated genes, were categorised as small ORFs. Homing endonuclease genes (HEGs) in introns were predicted based on similarity with other HEGs, and were not required to start with a methionine codon and stop codons were admitted inside the genes.

InterProscan (Hunter et al. 2009) was used to search for protein signatures in ncORFs. The small and large subunit (*rns*, *rnl*) ribosomal RNA (rRNA) genes were identified with BLAST using genes from closely related species. The program tRNAscan-SE (Lowe and Eddy 1997) was used to identify the tRNA genes. The *H. irregulare* mt genome with annotations have been submitted to GenBank, accession number KF957635.

The frequency of codon usage was calculated for core genes, exchangeable genes and small ORFs and differences were studied with correspondence analysis (COA) using CodonW (Peden 1999). Small ORFs with similar codon usage as conserved genes were categorised as small hypothetical ORFs and were subjected to BLAST analysis against the NCBI nucleotide database. Eight genes from the nuclear genome of *H. irregulare* [F-type ATPase alpha (protein ID 35948), F-type ATPase b (147326), cytochrome c oxidase assembly protein 11 (383265), cytochrome c oxidase assembly protein 15 (34965), translation elongation factor 1a (406970), glyceraldehyde 3-phosphate dehydrogenase (419475), heat shock protein 90 (311260) and calmodulin (148960)] were retrieved as reference genes and included in the COA analysis. Differences in codon usage between genes were visualised by plotting the position of each gene on the resulting COA-axis 1 and 2.

Anticodons of the 26 tRNAs and the concatenated codon usage of the annotated genes were compared. Artemis (Rutherford et al. 2000) and DNA plotter (Carver et al. 2009) were used to visualise and calculate the mt genome, GC content, GC skew and cumulative GC skew.

### Basidiomycete mitochondrial genome comparison

Nineteen thoroughly annotated Basidiomycete mt genomes present at the time of the study were retrieved from NCBI and EMBL (Table 1) (Paquin et al. 1997; Wang et al. 2008; Formighieri et al. 2008; Haridas and Gantt 2010; Stone et al. 2010; Yoon et al. 2012; Costa et al. 2012; Zhao et al. 2013; Ferandon et al. 2013; Hegedusova et al. 2014). The mt genome content of each species was divided into the following structural categories: (1) Electron transport and ATP synthesis genes, (2) rRNA genes, (3) tRNA genes, (4) Intergenic regions (without repetitive regions), (5) Repetitive regions, (6) Introns (without intronic ORFs), (7) Intronic ORFs, (8) ncORFs, (9) Putative plasmid genes and (10) The ribosomal protein gene *rps3*. Sequence lengths were calculated for each category using Artemis. In order to get comparable data between the species, all ncORFs were defined as ORFs with a length over a limit where the probability of finding such an ORF by chance was equal to finding a 420 bp ORF in *H. irregulare*, given the base composition of the species. Repeats were detected in the species with the program Repseek (Achaz et al. 2007) using a cutoff *p* value of 0.01. Some additional annotation of the intronic ORFs was done in *Cryptococcus neoformans, Phakopsora meibomiae, P. pachyrhizi, Trametes cingulata, Tilletia indica, T. walkeri*, *Ustilago maydis, Phlebia radiata, Lentinula edodes, M. roreri*, *Cantharellus cibarius, F. velutipes, A. bisporus*, and *Ganoderma lucidum.* The median differences of mt genome lengths between the Agaricomycete group and the other Basidiomycetes were tested with the Wilcoxon rank-sum test with the null hypothesis no difference in length.

**Table 1:** The 20 annotated Basidiomycete mitochondrial genomes used in the current study

Species	Class	Order	Accession number
*Pleurotus ostreatus*	Agaricomycetes	Agaricales	NC_009905
*Schizophyllum commune*	Agaricomycetes	Agaricales	NC_003049
*Moniliophthora perniciosa*	Agaricomycetes	Agaricales	NC_005927
*Moniliophthora roreri*	Agaricomycetes	Agaricales	NC_015400
*Lentinula edodes*	Agaricomycetes	Agaricales	NC_018365.1
*Flammulina velutipes*	Agaricomycetes	Agaricales	NC_021373
*Agaricus bisporus*	Agaricomycetes	Agaricales	JX271275
*Heterobasidion irregulare*	Agaricomycetes	Russulales	KF957635
*Trametes cingulata*	Agaricomycetes	Polyporales	NC_013933
*Phlebia radiata*	Agaricomycetes	Polyporales	NC_020148
*Ganoderma lucidum*	Agaricomycetes	Polyporales	HF570115
*Cantharellus cibarius*	Agaricomycetes	Cantharellales	NC_020368
*Cryptococcus neoformans* var. *grubii*	Tremellomycetes	Tremellales	NC_004336
*Ustilago maydis*	Ustilaginomycetes	Ustilaginales	NC_008368
*Tilletia indica*	Exobasidiomycetes	Tilletiales	NC_009880
*Tilletia walkeri*	Exobasidiomycetes	Tilletiales	NC_010651
*Jaminaea angkoriensis*	Exobasidiomycetes	Microstromatales	NC_023248
*Phakopsora meibomiae*	Urediniomycetes/pucciniomycetes	Uredinales	NC_014352
*Phakopsora pachyrhizi*	Urediniomycetes/pucciniomycetes	Uredinales	NC_014344
*Rhodotorula taiwanensis*	Urediniomycetes	Sporidiales	HF558455

## Results

### Mitochondrial genome structure and annotation of *H. irregulare*

**Fig. 1:**
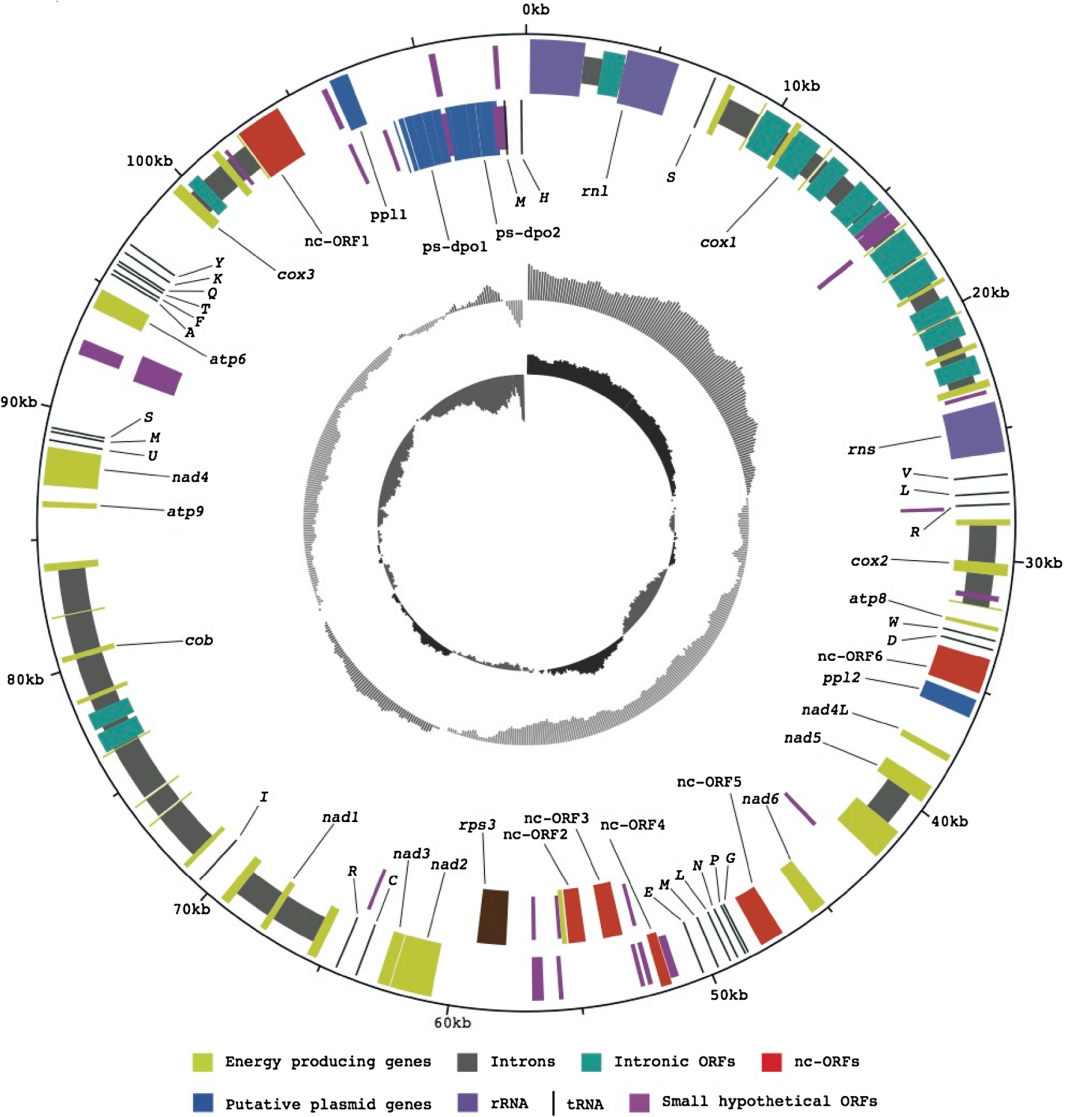
Map of the *Heterobasidion irregulare* mitochondrial genome. The *boxes* in *light green* are the energy producing genes, *rps3* is *brown*, the *turquoise boxes* are the intronic genes, *black lines* represent tRNAs, the non-conserved ORFs are *red-brown* and the *blue* are the putative plasmid and pseudo-*dpo* genes. The different rings, from the outside and in: (i) genes in the clockwise direction. (ii) Genes in the anticlockwise direction. (iii) GC content. (iv) GC skew

The circular 114, 193 bp long mt genome of *H. irregulare* had a mean GC content of 22.8 %. Fifteen protein coding core genes, 28 RNA genes, six ncORFs, 36 small hypothetical ORFs, 14 intronic ORFs, two putative plasmid genes, two putative *dpo* pseudogenes and one partial gene were identified (Fig. 1).

Seven out of the 15 core genes were predicted to encode proteins belonging to the NADH dehydrogenase complex (*nad1, nad2, nad3, nad4, nad4L, nad5, nad6*), one gene product belonged to the cytochrome bc1 complex (*cob*), three to the cytochrome c oxidase complex (*cox1, cox2, cox3*) and three to the ATP synthase complex (*atp6, atp8, atp9*). One additional partial *nad2* gene was also identified. The last core gene was predicted to encode the ribosomal small subunit protein *3* (*rps3*), which is involved in ribosome assembly. The identified RNA genes included the small and large subunit rRNA genes (*rns* and *rnl*) and 26 tRNAs. Two ORFs with similarity to each other (*E* value 6e^−17^) were annotated as putative plasmid genes (*ppl1* and *ppl2*), since they show similarity with putative plasmid proteins from *P. ostreatus* and *M. perniciosa* (*E* values 3e^−12^ and 3e^−7^). Two putative plasmid pseudo-B-type DNA polymerase genes (*ps*-*dpo1* and *ps*-*dpo2*) containing frame shifts were found next to one of the putative plasmid genes. In five of the six large ncORFs (ncORF1-6), transmembrane regions were found. Four of the ncORFs were located adjacent to each other, while two additional ncORFs were neighbouring putative plasmid genes (Fig. 1). The ncORFs had no similarity to each other.

In two regions, genes were predicted to be transcribed on the complementary strand compared to the majority of genes: one region with two of the four ncORFs adjacent to each other, the partial *nad2* gene and *rps3.* The other complementary strand region contains the two putative pseudo-*dpo* genes. The cumulative GC skew has a minimum value upstream of the *rnl* gene and is therefore considered as the putative location of the origin of replication for the *H. irregulare* mt genome (Touchon et al. 2005; Formighieri et al. 2008).

### Introns and intronic ORFs in *H. irregulare*

The 24 introns found were unevenly distributed among the core genes: nine in *cox1*, two in *cox2*, two in *cox3*, seven in *cob*, two in *nad1*, one in *nad5* and one in *rnl* (Fig. 1). In ten of these introns, 14 intronic ORFs were found. Ten were found in the introns of *cox1,* one in *cox3,* two in *cob* and one in *rnl*. There were as many as three intronic ORFs in intron four of *cox1,* whereof one was a putative pseudo-intronic ORF since it contained frame shifts. BLAST searches showed that the intronic ORFs were predicted to encode conserved homing endonuclease genes (HEGs, *E* values between 2e^−30^ and 1e^−132^). All but one intronic ORF contained one of the two HEG motifs: LAGLIDADG or GIY-YIG. The best BLAST hit of the HEGs was always a HEG in a homologous core gene intron but not necessary in a phylogenetically closely related fungus. Eight of the intronic ORFs were found in the same reading frame as the upstream exon, ten did not start with the typical methionine codon and three had internal stop codons in conserved sequences.

### The tRNAs and codon usage in *H. irregulare*

In total, 26 genes for tRNA was identified, clustered in nine regions. All but two tRNAs were clustered with two to six tRNAs in each group. Two tRNAs located between a pseudo-*dpo* gene and *rnl* were transcribed on the opposite strand (Fig. 1). For every amino acid there was one or more corresponding tRNA. However, the most used codons did not always have a corresponding tRNA in the mt genome even when the wobbling rule was taken into account (Crick 1966), (http://www.ncbi.nlm.nih.gov/books/NBK21424/). The following codons were among the most frequently used but did not have the corresponding tRNA in the mt genome: GCT (Ala), GGT (Gly), ATA (Ile/Met), CCT (Pro), TCT (Ser), ACT (Thr) and GTT (Val).

Codon usage was analysed for all core and exchangeable genes including all small ORFs. There was a strong codon bias towards using AT-rich codons in all genes and the small ORFs. The tRNAs, on the other hand, did not show the same bias towards AT-rich anticodons. Instead, the 5′ base of the anti codons strongly favoured primarily a T and secondarily a G: 22 of the 25 anticodons started with either a T or a G.

**Fig. 2:**
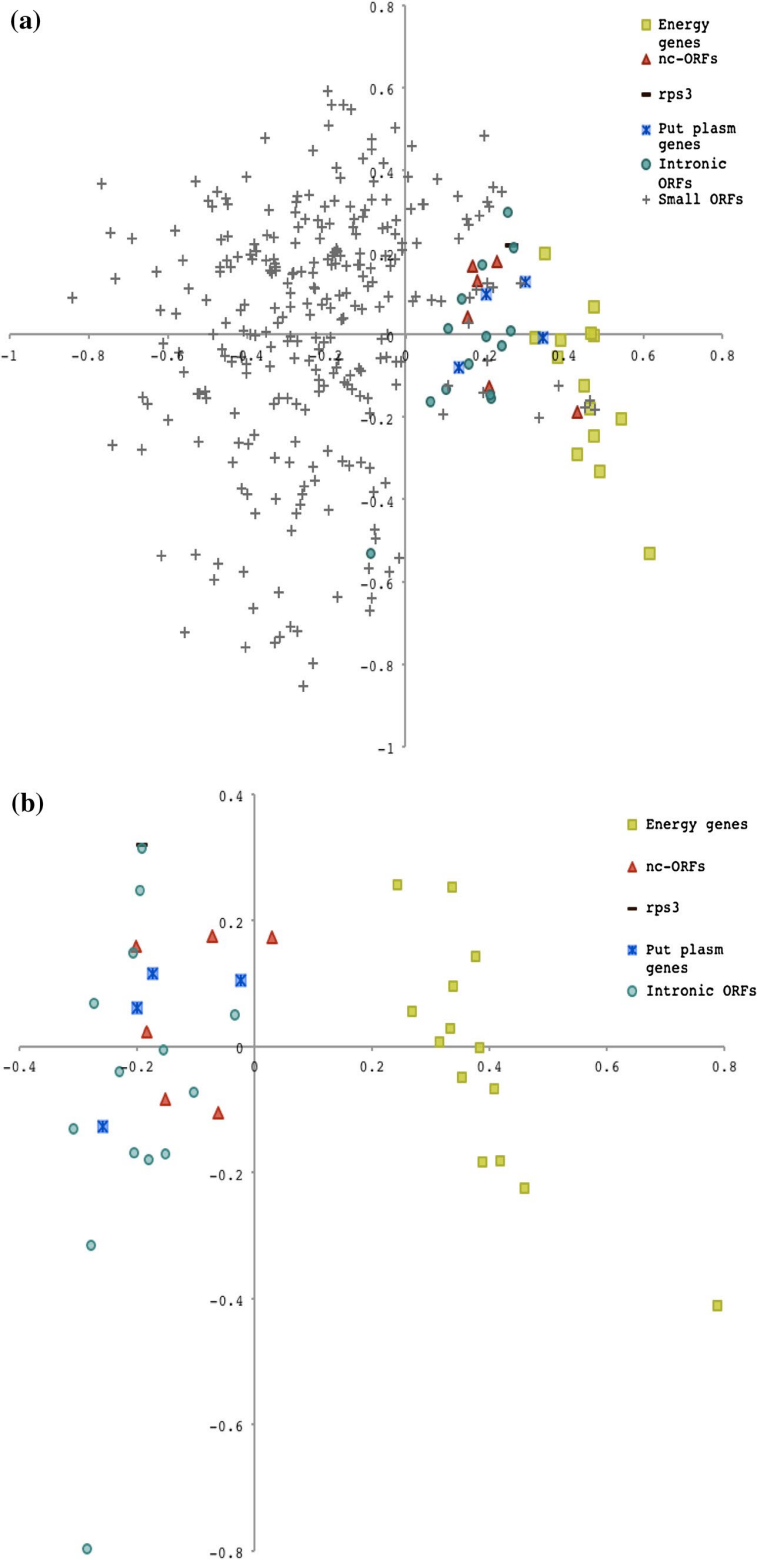
Correspondence analysis of codon usage of mitochondrial genes in *H. irregulare.* Correspondence analysis of codon usage was performed on annotated genes, putative genes including small ORFs (**a**) and without the small ORFs (**b**) identified in the mt genome of *H. irregulare*, using the program CodonW

Codon usage analysis with the annotated genes in the mt genome and eight nuclear genes clearly separated mt genome genes from nuclear genes along the first axis in a COA (data not shown). In the COA analysis of codon usage in annotated genes and the small ORFs, two groups were formed (Fig. 2a). One group consisted of all of annotated genes except one intronic ORF, the ncORFs along with some of the small ORFs. The remaining small ORFs were located in the second group on the negative side of axis 1.

**Fig. 3:**
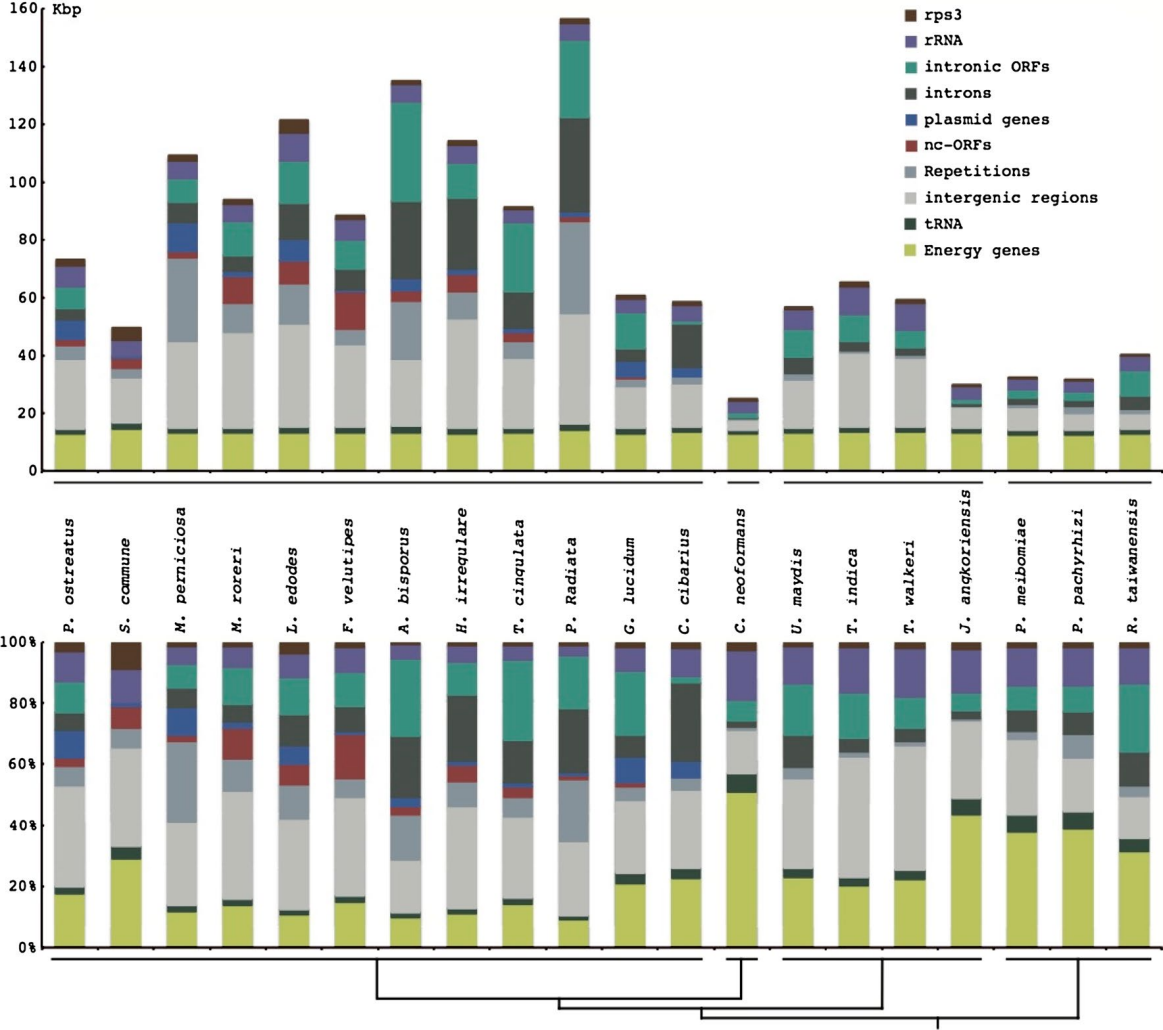
Comparison of Basidiomycete mitochondrial genome size differences, separated into different categories indicated in the diagram. The total DNA amount corresponding to each category in each genome (*top panel*) and the percentages of the categories for each mt genome (*bottom panel*) is plotted. A schematic representation of the taxonomic relationships of the included species is given

When performing the COA excluding small ORFs, two distinct groups were formed (Fig. 2b). One group consisted of genes predicted to encode proteins involved in energy production, while the other group consisted of genes that were not predicted to be involved in energy production. There were 11 codons that differed significantly in usage between the two groups: Leu UUA, Val GUA, Tyr UAU, Glu GAA (*p* < 0.01) and Phe UUC, Ile AUA, Gln CAA, Asp GAU, Ser UCA, Pro CCA, Arg AGA (0.01 < *p* < 0.05). Eight of these 11 codons had a corresponding tRNA in the mt genome (Fig. 3).

### Comparative analysis of Basidiomycete mitochondrial genomes

To analyse the relative contribution in size of different mt genome parts for Basidiomycetes, 19 mt genomes (Table 2) were re-annotated in a consistent way. This resulted in the identification of *rps3,* previously not reported, in the two *Tilletia* species. In addition, some of the exchangeable genes were in unusual positions. There were HEGs in intergenic areas in *T. indica, M. perniciosa,* and *G lucidum* and putative plasmid genes in introns in *T. cingulata* and *A. bisporus.* All the mt genomes including *H. irregulare* had the same set of core genes: *nad1, nad2, nad3, nad4, nad4L, nad5, nad6, cob, cox1, cox2, cox3, atp6, atp8, atp9, rps3,* *rns* and *rnl*. However, the *P. radiata* mt genome contained two *nad6* genes due to a 6,077 bp long duplication. Putative plasmid genes were found in all the 12 Agaricomycete mt genomes and ncORFs in 11 of them. There was a large variation in the number and positions of putative plasmid genes and ncORFs in the various mt genomes. In *M. perniciosa*, the six putative plasmid genes were clustered in the same location and five of them were within two inverted repeats typical for mt plasmids. There were three ncORFs in the same area (Formighieri et al. 2008). In the closely related species *M. roreri*, a 14 Kb area between *nad3* and *nad1* was found with five ncORFs and two putative plasmid genes (according to our criteria) (Costa et al. 2012). *Agaricus bisporus* had two large inverted repeats and each of them contained a remnant copy of a DNA polymerase gene (Ferandon et al. 2013). Between the inverted repeats there were also two putative plasmid genes and two ncORFs plus the genes *rps3, rnl, nad3* and *nad2.* There were three areas in A. bisporus where putative plasmid genes and ncORFs were clustered together. The putative plasmid gene in *F. velutipes* was clustered together with three ncORFs. In the *H. irregulare* mt genome, three of the four putative plasmid genes were clustered together with an ncORF. The four putative plasmid genes in *C. cibarius* were all clustered in the same area. In the other six Agaricomycete mt genomes, the putative plasmid genes and ncORFs were more evenly distributed. The amount of repetitive areas differed between the species, whereof *M. perniciosa* and *P. radiata* were the most outstanding with a repetitive content of 26.5 and 20.3 %, respectively. The repetitive regions were also the most variable areas with a coefficient of variation of 1.27 followed by introns (without intronic ORFs), intronic ORFs, putative plasmid genes, ncORFs, *rps3* and intergenic regions (Table 3). The energy production genes had by far the smallest coefficient of variation (0.04). The nine largest mt genomes among the studied Basidiomycetes all belonged to the Agaricomycetes and all nine contained both introns with intronic ORFs and putative plasmid genes together with ncORFs. *P. radiata* had the largest mt genome (156,348 bp) and the largest areas of intergenic regions (69,951 bp), introns without intronic ORFs (32,536 bp) and repetitive sequences (31,805 bp) mostly found in the intergenic regions. The *A. bisporus* mt genome (135,005 bp) had the highest number of introns (46) and the largest areas of intronic ORFs (34,057 bp), whereas *L. edodes* (121,394 bp) had larger areas of intergenic regions (49,586 bp), ncORFs (7,264 bp) and putative plasmid genes (7,264 bp) and *H. irregulare* (114,193 bp) had larger areas with introns (24,921 bp). *M. perniciosa* (109,103 bp) had the largest areas with putative plasmid genes (7,264 bp) and also large areas with intergenic (58,832 bp) and repetitive regions (28,951 bp) in comparison with the other mt genomes. *F. velutipes* (88,508 bp) had the largest areas of ncORFs (12,807 bp). The Ustilaginomycete and Exobasidiomycete species had medium mt genome sizes (57–65 kbp), with the exception for *J. angkoriensis,* the three Urediniomycetes species and *C. neoformans* all had small mt genome sizes (<40 kbp). The Agaricomycete genomes were significantly larger than other Basidiomycetes (*p* = 0.00024).

**Table 2:** Characteristics of the Basidiomycete mitochondrial genomes used in the current study

Species	Length (bp)	GC content (%)	ncORF cut off (bp)	Repetitive areas (%)	tRNA	Intronic ORFs	Introns	ncORFs	Plasmid genes
*P. ostreatus*	73,242	26.4	460	6.51	24	9	12	2	3
*S. commune*	49,704	21.9	410	6.48	27	0	0	4	1
*M. perniciosa*	109,103	31.9	540	26.54	26	9	13	8	6
*M. roreri*	93,722	27.6	480	10.72	26	12	12	9	2
*L. edodes*	121,394	30.7	525	11.37	28	15	19	7	3
*F. velutipes*	88,508	16.5	355	6.00	26	10	10	7	1
*A. bisporus*	135,005	29.1	500	14.78	35	37	46	4	6
*H. irregulare*	114,193	22.8	420	8.17	26	14	24	6	2
*T. cingulata*	91,500	24.5	440	6.24	25	28	26	4	1
*P. radiata*	156,348	31.2	530	20.34	28	28	37	2	1
*G. lucidum*	60,630	26.7	465	4.39	28	14	13	1	3
*C. cibarius*	58,656	26.8	470	4.20	26	13	13	0	4
*C. neoformans*	24,874	35.0	600	1.00	21	2	2	0	0
*U. maydis*	56,814	31.2	530	3.71	23	12	14	0	0
*T. indica*	65,147	28.9	500	1.54	24	11	11	0	0
*T. walkeri*	59,352	28.8	500	1.30	24	7	9	0	0
*J. angkoriensis*	29,999	32.2	545	0.75	22	2	2	0	0
*P. meibomiae*	32,520	34.9	595	2.76	24	3	5	0	0
*P. pachyrhizi*	31,825	34.6	590	7.75	24	3	5	0	0
*R. taiwanensis*	40,392	41.0	730	3.30	23	8	13	0	0

**Table 3:** Variation in length of the different categories and the repetitive regions, in the form of standard deviation and coefficient of variation

Category	Mean	Standard deviation	Coefficient of variation
Repeat regions	7,354.80	9,365.86	1.27
Introns^a^	9,272.95	9,372.85	1.01
Intronic ORFs^a^	10,852.26	8,944.20	0.82
Plasmid genes^a^	3,685.67	3,008.70	0.82
ncORFs^a^	4,833.82	3,790.91	0.78
rps3	1,426.80	1,084.65	0.76
Intergenic regions	28,200.15	18,721.15	0.66
rRNA	6,021.95	1,802.27	0.30
tRNA	1,911.30	230.84	0.12
Energy genes	13,102.05	518.13	0.04

^a^Species missing this category were excluded from the analysis

## Discussion

The 114 kb long *H. irregulare* mt genome is one of the largest sequenced and annotated fungal mt genomes. *Heterobasidion irregulare* belongs to the class Agaricomycete that contains a majority of the species with the largest fungal mt genomes known to date. All of the largest Agaricomycete mt genomes have long regions with intergenic areas, ncORFs and putative plasmid genes whereas some also have long regions with introns and intronic ORFs. Variable mt genome sizes have previously been attributed to intergenic regions, introns and in some cases extensive tandem repeat arrays or stem loop motifs (Burger et al. 2003). Our observations show that variation in exchangeable gene content and repeat regions are also important contributors to differences in mt genome size.

Many fungal and plant species have mt plasmid DNA integrated in their mt genomes, despite the fact that no genes coding for integrase activity has been found in mt plasmids so far (Cahan and Kennell 2005). All the mt genomes of Agaricomycetes contain putative plasmid genes and ncORFs, with the exception for *C. cibarius* where the mt genome lacks ncORFs. However, both putative plasmid genes and ncORFs are absent in the other studied Basidiomycete classes. Our data suggests that the presence of ncORFs in mt genomes is somehow linked to mt plasmids based on the following observations: (1) The simultaneous occurrence of putative plasmid genes and ncORFs in mt genomes. (2) NcORFs and putative plasmid genes are often clustered in several of the mt genomes. (3) The ncORFs have the same preferential codon usage as the putative plasmid genes in *H. irregulare*. Given the coexistence of ncORFs and putative plasmid genes in Agaricomycetes, the lack of ncORFs in the *C. cibarius* mt genome most likely represents a loss of ncORFs in the ancestor of *C. cibarius*. The apparent connection may suggest that the ncORFs originate from an mt plasmid, or alternatively that mt plasmids may enable integration of non-mitochondrial DNA into the mt genome. The different patterns of codon usage in the putative plasmids and ncORFs, compared to the core genes, may indicate separate evolutionary origins of these two classes of genes. Since putative plasmid genes occurred exclusively in the Agaricomycetes, we hypothesise that the common ancestor of the Agaricomycetes acquired the mt plasmids. The acquisition might have been initiated by the uptake of a plasmid into the mitochondria in the Agaricomycete ancestor, which was subsequently integrated into the mt genomes at several, independent occasions. A single integration event in the Agaricomycete ancestor is less likely since there is a considerable variation in the number and positioning of the putative plasmid genes among the Agaricomycete species.

HEGs have previously been proposed to go through a cyclical model of invasion, degeneration and loss where the HEGs only are selected for during the invasion phase. Nonetheless, some HEGs have been reported to persist over longer evolutionary times (Goddard and Burt 1999; Gogarten and Hilario 2006).

All Basidiomycete mt genomes except *S. commune* contained introns and intronic ORFs. However, the length of these regions varied largely among species with the largest regions in the Agaricomycete species. In this study, we found that a particular HEG of *H. irregulare* always showed the highest similarity to an HEG in an intron of a homologous core gene of another species, although it was not necessarily the closest related species that contained the most similar HEGs. This is in accordance with previous findings that group I introns have a preference to be inserted into highly conserved genes, such as *cox1* and *cob* (Paquin et al. 1997). Furthermore, between species, the intron movement nearly always occurs into the homologous gene site (Paquin et al. 1997; Haugen et al. 2005). Phylogenetic analyses suggest that horizontal gene transfer has occurred frequently but the mechanism of how the mobile introns are transferred between species barriers is not known (Goddard and Burt 1999; Wang et al. 2008). In *H. irregulare*, the intronic ORFs had a different preferential codon usage than the energy genes. The most likely explanation to this is that the intronic ORFs have a different evolutionary origin than the energy genes, possibly acquired through horizontal gene transfer. Alternatively, differences in expression levels can also result in differences in preferred codon usage. However, no study of intronic ORF gene expression is reported from *H. irregulare*.

Recently, an invasion of HEGs and self-splicing introns was shown to explain the large mt genome size of *Rhynchosporium agropyri* and *R. secalis* (Torriani et al. 2014). Similar expansions of mobile introns are reported from other ascomycete fungi including *Fusarium* spp. (Al-Reedy et al. 2012), *Penicillium* spp. and *Aspergillus* spp. (Joardar et al. 2012). The reoccurring internal stop codons and frame shifts and the varying number of HEGs in the studied species suggest that HEG and intron expansions can contribute to large mt genome sizes also in Basidiomycetes as previously reported for ascomycete fungi.

The presence of long ncORFs in fungal mt genomes with no similarity to genes in other species is common, and can contribute to a large mt genome size (Al-Reedy et al. 2012). However, it is difficult to confirm that these are authentic genes in the absence of homologous genes from other species. The ncORFs found in *T. cingulata* do not utilise the same codons as the core genes and were proposed not to correspond to authentic genes (Haridas and Gantt 2010). Since stop codons are AT rich, the probability for ncORFs larger than 420 bp to occur by chance in a low GC content mt genome such as in *H. irregulare* is less than 7.5e^−15^. The fact that the ncORFs displayed the same preferential codon usage as conserved exchangeable genes, and the presence of several predicted transmembrane domains, suggests that the identified ncORFs are indeed authentic genes that could possibly originate from mt plasmids. On the other hand, 14 ncORFs with the same preferential codon usage as the core genes were found in *M. perniciosa* (Formighieri et al. 2008), which prompted the authors to suggest that the ncORFs represent functional genes that originate from the α-proteobacterial ancestor. However, the ncORF in the *G. lucidum* mt genome found in this study was not expressed in a recent transcriptome analysis (Li et al. 2013).

We identified two independent factors as the driving forces for large mt genomes among Basidiomycetes. The first factor was the HEGs in the introns that are able to make the introns mobile and able to spread into intronless genes. The second factor was plasmid regions that are integrated in mt genomes, possibly leaving plasmid gene remnants in the form of ncORFs and larger intergenic areas. The *H. irregulare* isolate in this study was one of the parents in the interspecies crossing study that showed a connection between the mitochondria and virulence (Olson and Stenlid 2001). Possible factors that could have an influence on virulence are probably not the core genes of the mt genome but rather the exchangeable parts. We are now well positioned to continue this investigation by comparing the mt genome of *H. irregulare* with *H. occidentale,* the other parent in the hybrid study (Olson and Stenlid 2001).
